# Novel Genes and Genetic Loci Associated With Root Morphological Traits, Phosphorus-Acquisition Efficiency and Phosphorus-Use Efficiency in Chickpea

**DOI:** 10.3389/fpls.2021.636973

**Published:** 2021-05-28

**Authors:** Mahendar Thudi, Yinglong Chen, Jiayin Pang, Danamma Kalavikatte, Prasad Bajaj, Manish Roorkiwal, Annapurna Chitikineni, Megan H. Ryan, Hans Lambers, Kadambot H. M. Siddique, Rajeev K. Varshney

**Affiliations:** ^1^Center of Excellence in Genomics and Systems Biology, International Crops Research Institute for the Semi-Arid Tropics (ICRISAT), Hyderabad, India; ^2^The UWA Institute of Agriculture, The University of Western Australia, Perth, WA, Australia; ^3^State Agricultural Biotechnology Centre, Centre for Crop and Food Innovation, Food Futures Institute, Murdoch University, Murdoch, WA, Australia

**Keywords:** chickpea, genome-wide association study, phosphorus-acquisition efficiency, phosphorus-use efficiency, root traits, genetic mapping

## Abstract

Chickpea—the second most important grain legume worldwide—is cultivated mainly on marginal soils. Phosphorus (P) deficiency often restricts chickpea yields. Understanding the genetics of traits encoding P-acquisition efficiency and P-use efficiency will help develop strategies to reduce P-fertilizer application. A genome-wide association mapping approach was used to determine loci and genes associated with root architecture, root traits associated with P-acquisition efficiency and P-use efficiency, and any associated proxy traits. Using three statistical models—a generalized linear model (GLM), a mixed linear model (MLM), and a fixed and random model circulating probability unification (FarmCPU) —10, 51, and 40 marker-trait associations (MTAs), respectively were identified. A single nucleotide polymorphism (SNP) locus (Ca1_12310101) on Ca1 associated with three traits, i.e., physiological P-use efficiency, shoot dry weight, and shoot P content was identified. Genes related to shoot P concentration (NAD kinase 2, dynamin-related protein 1C), physiological P-use efficiency (fasciclin-like arabinogalactan protein), specific root length (4-coumarate–CoA ligase 1) and manganese concentration in mature leaves (ABC1 family protein) were identified. The MTAs and novel genes identified in this study can be used to improve P-use efficiency in chickpea.

## Introduction

Phosphorus (P) is an essential nutrient for crop production. Using spatially explicit global maps for more than 100 crops, MacDonald et al. (2011) reported that 29% of the global cropland area is P deficient, while 71% has surplus P. For example, 42% of field soil in India is low in P, 38% is medium and 20% is high (Motsara, 2002). Excessive P fertilizer and manure application in industrialized countries have led to low P-use efficiency (PUE), with surplus P retained in soil as residual P (Syers et al., 2008) or lost to the environment where it is causing significant water quality problems. Globally 51–86% more P input will be needed for sustainable crop production by 2050 (Mogollón et al., 2018), unless we work toward more P-efficient crops and cropping systems (Cong et al., 2020).

Chickpea (*Cicer arietinum* L.), is one of the most important grain legumes cultivated by smallholder farmers in more than 50 countries. Advances in chickpea genomics in the last decade have made large-scale genomic resources available to the chickpea research community including molecular markers (Thudi et al., 2011; Hiremath et al., 2012), genetic maps (Nayak et al., 2010), genome sequences (Varshney et al., 2013b), and resequencing of several germplasm lines (Thudi et al., 2016a, b; Varshney et al., 2019). These resources have improved our understanding of both abiotic (Varshney et al., 2014) and biotic stress tolerance in chickpea (Sabbavarapu et al., 2013) and enabled fine mapping of traits (Jaganathan et al., 2015; Kale et al., 2015). Furthermore, the resources have been successfully used to develop new varieties with enhanced tolerance or resistance (see Varshney et al., 2013a; Mannur et al., 2019; Bharadwaj et al., 2020; Roorkiwal et al., 2020). Twelve chickpea genotypes with well-known responses under drought and irrigation were evaluated for profuse root length density (RLD) in surface soil and root dry weight (RDW) and root:shoot ratio (RSR) in deeper soil layers (Purushothaman et al., 2017). This study revealed that drought stress increased RLD below 300 mm soil depth, deep RDW, and RSR, but decreased root diameter. Moisture-conservation practices and optimum P levels to enhance PUE were reported recently in chickpea grown in vertisols in central India (Chaudhary et al., 2018). In soybean, root length is positively correlated with P accumulation in well-watered and water-stressed conditions (He et al., 2017). Phenotypic plasticity and genetic variability in root architectural traits of chickpea and their role in drought tolerance using a novel semi-hydroponic system have been reported (Chen et al., 2017). A recent study revealed that root system plasticity affects P acquisition efficiency, PUE, and photosynthetic PUE in 266 chickpea genotypes (Pang et al., 2018c). Root exudates like carboxylate enable P acquisition from the soils which are low in available P. Manganese concentration in mature chickpea leaves is positively correlated with the amount of rhizosheath carboxylates, offering an easily measurable proxy for assessing rhizosheath carboxylates in 100 chickpea genotypes grown under low P availability (Pang et al., 2018a). In the context of global climate change, Pang et al. (2018b) summarized the factors affecting PUE and enhancing P-acquisition efficiency in legumes, and key areas for future research. Understanding the genetics of these traits, identification of genomic regions, molecular markers and or marker trait associations for PUE efficiency related traits will help improving these traits through marker-assisted selection or genomics-assisted breeding.

Various type of molecular markers have been used to establish marker-trait associations (MTA) in chickpea. For instance, 1,072 Diversity Arrays Technology (DArTs), 651 single nucleotide polymorphisms (SNPs), 113 gene-based SNPs, and 36 simple sequence repeats (SSRs) were used to establish 312 MTAs for drought- and heat-tolerance related traits (Thudi et al., 2014). SNPs were used extensively for association studies in chickpea due to their abundance and amenability for high-throughput genotyping (Diapari et al., 2014; Jadhav et al., 2015; Upadhyaya et al., 2016; Varshney et al., 2019; Sab et al., 2020).

In legumes, genome-wide association studies (GWAS) are gaining momentum. A recent study reported two SNP markers tightly linked to seed iron (Fe) and one to seed zinc (Zn) concentration in lentils (*Lens culinaris* Medik.) (Khazaei et al., 2017). Another study reported 159 quantitative trait nucleotides (QTNs) and 52 candidate genes associated with the photosynthetic response to low-P stress in soybean [*Glycine max* (L.) Merr.] (Lü et al., 2018). In chickpea, association mapping between SNP markers and seed copper (Cu), P, and potassium (K) concentrations identified eight SNPs associated with variation in three nutrients in more than two environments (Ozkuru et al., 2018). Similarly, seed mineral concentration in pea (*Pisum sativum* L.; Gali et al., 2019), Fe chlorosis in soybean; Mamidi et al., 2014; Assefa et al., 2020), and Fe bioavailability in cooked dry beans (*Phaseolus vulgaris* L.; Katuuramu et al., 2018) have been mapped. Novel genes involved in the accumulation of P in *Lotus japonicas* have been reported using GWAS analysis (Giovannetti et al., 2019).

In view of above, this study was conducted to undertake GWAS analysis to identify MTAs for (i) root architectural traits evaluated in a high-throughput semi-hydroponic root phenotyping platform, and (ii) root morphological and physiological traits related to P-acquisition efficiency and P−use efficiency under low P supply. This is the first study that reports genomic regions associated with above mentioned traits by using three different models, namely fixed and random model circulating probability unification (FarmCPU), mixed linear model (MLM), and generalized linear model (GLM) in the GAPIT-R package. The reference genome has been used to identify the candidate genes in the identified MTAs associated with above traits.

## Materials and Methods

### Germplasm Lines and Phenotyping

The chickpea reference set (Upadhyaya et al., 2008) comprising of 300 diverse accessions (267 landraces, 13 advanced lines and cultivars, 7 wild *Cicer* accessions, and 13 accessions with unknown biological status) was used for phenotyping datasets as following: (i) 270 genotypes of the reference set were evaluated for 30 root architectural traits in a semi-hydroponic phenotyping system (Chen et al., 2017); (ii) Two hundred and sixty-six genotypes (including 255 from the chickpea reference set along with 11 Australian chickpea cultivars (Ambar, Almaz, Neelam, Genesis 079, Genesis 090, Genesis 509, Genesis 836, Genesis Kalkee, PBA Boundary, PBA Slasher and PBA Striker) were evaluated for P-acquisition efficiency and P−use efficiency with P supplied as insoluble FePO_4_ (Pang et al., 2018c); and (iii) a selected subset of 100 chickpea genotypes of the reference set—showing visual differences in plant size or leaf symptoms of P deficiency—phenotyped for shoot/root morphological and physiological traits to understand the relative roles of root morphology and physiology in P-acquisition efficiency (Pang et al., 2018a, c).

### Genotyping and Determining Population Structure

The SNP dataset, based on whole-genome resequencing data on the reference dataset was filtered for missing values (≥20%) and minor-allele frequency <5% using vcftools and imputed by BEAGLE-5.0 (Browning and Browning, 2016; Varshney et al., 2019). As the number of genotypes were different in data sets, Population structure was separately determined for the chickpea reference set and 91 genotypes (from the 100 selected subset of 199 genotypes) using ADMIXTURE v1.3.0 (Zhou et al., 2011).

### Genome-Wide Association Analysis

#### GWAS for Root Traits and Seven P-Acquisition Efficiency Traits

To identify significant MTAs and avoid spurious associations of the 270 genotypes mentioned above, 233 genotypes (with phenotyping data and genotyping data) were considered for GWAS analysis. GWAS analysis was performed using 698,183 SNPs (SNP calls obtained based on aligning 233 genotypes to reference genome CDC Frontier) and phenotyping data for 37 traits (i.e., 30 root traits and seven P-acquisition efficiency traits). MTAs were determined using three models, namely fixed and random model circulating probability unification (FarmCPU), mixed linear model (MLM), and generalized linear model (GLM) in the GAPIT-R package (Lipka et al., 2012).

#### GWAS for Biochemical Traits/Proxy Traits for PUE

A total of 706,865 SNPs and phenotyping data generated on 91 chickpea germplasm lines was for analysis in GAPIT-R using FarmCPU and MLM models to determine MTAs for biochemical traits.

The Bonferroni correction threshold of 7.07E-08 was used to avoid spurious associations. The genes involving significant SNP markers were aligned against the NCBI non-redundant (nr) protein database taxon Viridiplantae using BLASTX, to obtain functional annotations. GO and KEGG (Kyoto Encyclopedia of Genes and Genomes) pathway identification were conducted on these sequences in the KEGG pathways in-built in BLAST2GO. The SNPEff- 4.3T open source program was used for variant annotation and prediction of significant SNP effects.

## Results

### Population Structure and Genome-Wide Association Study (GWAS)

Using genome-wide SNP data for 233 chickpea genotypes (with both genotypic and phenotypic data), three subpopulations were identified (Supplementary Figures 1A,B) using ADMIXTURE. Similarly, in the smaller subset of 91 genotypes, three subpopulations were identified (Supplementary Figures 1C,D). Three statistical models—GLM, MLM, and FarmCPU—enabled identification of 10, 51, and 40 MTAs, respectively (Table 1 and Supplementary Table 1) after applying Bonferroni and FDR corrections. Spurious MTAs were excluded by examining Q/Q plots. Forty-two of the MTAs identified by the GLM and MLM models were robust (>10% of the phenotypic variation explained); the phenotypic variation explained by the MTAs identified in the FarmCPU model was not computed. The GLM model identified MTAs for four traits (root growth rate, root mass density, specific root length, and shoot P concentration); this low number of traits could be due to the many spurious MTAs (Supplementary Figure 2). The FarmCPU model identified MTAs for the most traits, followed by the MLM model.

**TABLE 1:** Summary of marker trait associations identified using three statistical models namely GLM, MLM and FarmCPU.

Trait	Code	GLM			MLM			FarmCPU	
		No. of MTAs	*p*-value	R2	No. of MTAs	*p*-value	*R*^2^	No. of MTAs	*p*-value
Average branch length	(ABL; cm per branch)	–	–	–	2	5.92 × 10^–8^ to 3.69 × 10^–8^	5.01–8.24	1	5.02 × 10^–8^
Branch density	(BD; cm^–1^ taproot)	–	–	–	–	–	–	2	6.41 × 10^–10^ to 2.67 × 10^–10^
Branch intensity	(BI; cm^–1^ root)	–	–	–	–	–	–	1	7.51 × 10^–9^
Root diameter s2	(RD_s2; mm)	–	–	–	–	–	–	1	5.14 × 10^–9^
Subsoil root diameter	(RD_sub; mm)	–	–	–	–	–	–	1	7.2 × 10^–14^
Topsoil root diameter	(RD_top; mm)	–	–	–	–	–	–	1	7.46 × 10^–12^
Root growth rate	(RGR; cm d^–1^)	1	2.2 × 10^–8^	13.13	12	5.62 × 10^–15^ to 1.9 × 10^–8^	9.2–11.78	–	–
Root length ratio	(RLR_top/sub)	–	–	–	–	–	–	1	1.06 × 10^–8^
Root length s2	(RL_s2; cm)	–	–	–	–	–	–	1	1.59 × 10^–8^
Root mass	(RM; mg)	–	–	–	–	–	–	1	3.86 × 10^–8^
Root mass ratio	RMR	–	–	–	6	4.4 × 10^–25^ to 1.73 × 10^–8^	20.86–30.12	1	3.8 × 10^–8^
Root_2	Root diameter (mm)	–	–	–	1	1.77 × 10^–12^	7.52	–	–
Root tissue density	(RTD; mg cm^–3^)	2	2.88 × 10^–9^ to 6.73 × 10^–8^	14.23 to 16.95	–	–	–	2	7.18 × 10^–15^ to 4.58 × 10^–10^
Shoot dry weight	SDW (mg)	–	–	–	–	–	–	1	6.31 × 10^–9^
Specific root length	(SRL; m g^–1^ dry mass)	3	2.48 × 10^–9^ to 5.94 × 10^–8^	17.2 to 20.31	2	1.88 × 10^–10^ to 1.44 × 10^–9^	17.63	3	2.99 × 10^–11^ to 4.06 × 10^–9^
Taproot length zone1	(TRL_z1; cm)	–	–	–	1	7.15 × 10^–8^	13.59	1	7.15 × 10^–8^
Taproot length zone2	(TRL_z2; cm)	–	–	–	1	1.48 × 10^–11^	9.64	1	1.48 × 10^–11^
WUE	WUE (A/gs)	–	–	–	–	–	–	2	2.80 × 10^–11^ to 1.85 × 10^–8^
Total rhizo dry soil (g plant-1)	Total rhizo (g plant^–1^)	–	–	–	6	5.66 × 10^–17^ to 1.34 × 10^–11^	5–17.63	–	–
Phosphorous-utilization efficiency		–	–	–	13	4.45 × 10^–18^ to 8.41 × 10^–15^	20.23–26.16	1	2.13 × 10^–9^
Physiological P-use efficiency	PPUE (μmol g^–1^ P s^–1^)	1	5.46519E-08	11.95	–	–	–	–	–
Shoot phosphorous concentration	mg g^–1^	3	2.85 × 10^–9^ to 7.65 × 10^–8^	27.67 to 40.33	2	5.23 × 10^–9^ to 2.85 × 10^–9^	27.69–32.97	–	–
Shoot P content	Shoot P content	–	–	–	5	8.2 × 10^–9^ to 7.7 × 10^–8^	39.41.3	1	1.68 × 10^–13^
Carboxylate_2	Carboxylate conc (μmol g^–1^ root DW)	–	–	–	–	–	–	1	5.93 × 10^–8^
Ci	Ci	–	–	–	–	–	–	1	8.52 × 10^–10^
Citric_2	Citric (μmol plant^–1^)	–	–	–	–	–	–	1	1.42 × 10^–9^
Malonic (μmol plant^–1^)	Malonic (μmol plant^–1^)	–	–	–	–	–	–	1	4.82 × 10^–10^
Mn concentration in mature leaves	Mn_ML	–	–	–	–	–	–	7	6.08 × 10^–21^
P_ML	P_ML (mg g^–1^)	–	–	–	–	–	–	1	6.08 × 10^–21^
P_Pn	Pn_area (μmol m^–2^ s^–1^)	–	–	–	–	–	–	1	2.6 × 10^–8^
Pn_mass	Pn_mass (μmol g^–1^ s^–1^)	–	–	–	–	–	–	1	3.57 × 10^–9^
Rhizo-pH		–	–	–	–	–	–	2	1.72 × 10^–15^ to 2.24 × 10^–8^
Specific rhizosheath weight	(g g^–1^ root DW)	–	–	–	–	–	–	1	2.97 × 10^–8^
Total MTAs		10			51			40	

### GWAS Signals for Root Architectural Traits

Fifty-seven MTAs were identified for 19 root architectural traits phenotyped in our earlier studies (Chen et al., 2017; Pang et al., 2018c; Table 1). The FarmCPU model identified one significant MTA each in root depth zone 1 (TRL_z1; cm) and root depth zone 2 (TRL_z1; cm) on Ca3 and Ca6 for taproot length (i.e., root depth, z1 + z2), one significant MTA (Ca3_26114159) for root mass (RM, mg), two significant MTAs (Ca4_29694614 and Ca6_57970784) for branch density (BD; cm^–1^ taproot), and one significant MTA (Ca2_6340118) for branch intensity (BI; cm^–1^ root). No significant MTAs were detected for the above root traits in the GLM and MLM methods. Nevertheless, the MLM model identified six significant MTAs and FarmCPU identified one MTA for root mass ratio (RMR), and the MLM and FarmCPU models identified two significant MTAs (one each) for root length ratio (RLR_top/sub) and three significant MTAs (two and one, respectively) for average branch length (ABL; cm per branch). The FarmCPU model identified one significant MTA each for root diameter s2 (RD_s2; mm), subsoil root diameter (RD_sub; mm), and topsoil root diameter (RD_top; mm) on Ca6, Ca1, and Ca2, respectively. The FarmCPU model also identified two significant MTAs for water-use efficiency (WUE; A/g_*s*_) on Ca6 and Ca8. Based on the physical position of the SNP loci (MTAs) associated with different traits on Ca4, none of the MTAs was mapped in “*QTL-hotspot_a*” or “*QTL-hotspot_b*” that harbors several drought-tolerance-related root traits (Kale et al., 2015). The MTAs revealed key root traits for efficiently acquiring soil resources and adapting to drought and other abiotic stresses. Of the 13 significant MTAs (12 from MLM and one from GLM) identified for RGR (cm d^–1^), six (46.2%) were identified on Ca3 and three (23.1%) on Ca6. Six MTAs identified on Ca6 explained 3.6–10.0% of the phenotypic variation associated with five genes (Ca_08259, Ca_01151, Ca_01152, Ca_01156, and Ca_16553). Both statistical models (GLM and MLM) detected one significant MTA (Ca1_4716136), which explained higher phenotypic variation (11.8–13.1%) than the other MTAs (Supplementary Table 1). Eight significant MTAs were identified for specific root length (SRL, cm) in all three models; of these, one MTA on Ca7 (Ca7_3606123), explaining about 17.8% of the phenotypic variation, was consistent in all three models used for GWAS analysis. The MTA was associated with the Ca_03107 gene that encodes pectinesterase/pectinesterase inhibitor PPE8B. Six MTAs for total rhizosheath dry soil (g plant^–1^) were identified on Ca8 (3), Ca5 (1), Ca2 (1), and Ca1 (1).

### GWAS Signals for P-Acquisition Efficiency and PUE-Related Traits

**FIGURE 1:**
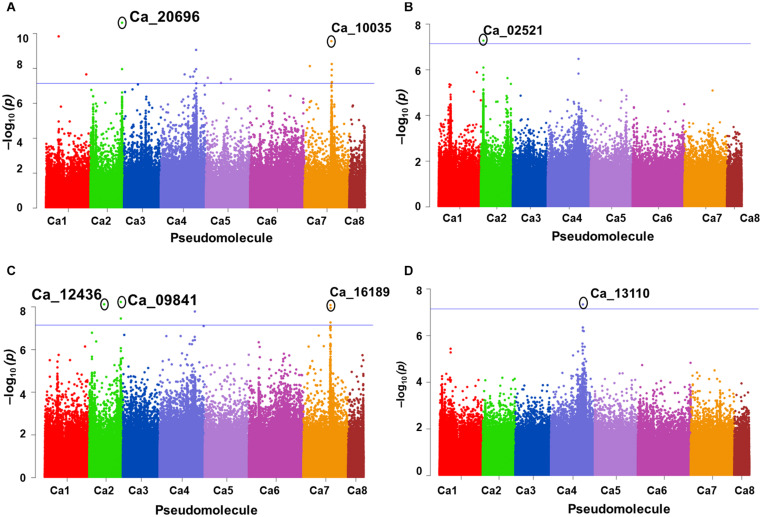
GWAS signals for phosphorus (P) -acquisition and P-use efficiency-related traits. **(A)** Shoot P concentration, **(B)** physiological P-use efficiency, **(C)** P-utilization efficiency, and **(D)** total shoot P content. Three different statistical models GLM, MLM, and FarmCPU were used to identify the MTAS. The significant MTAs were determined using Bonferroni correction (Table 1).

**FIGURE 2:**
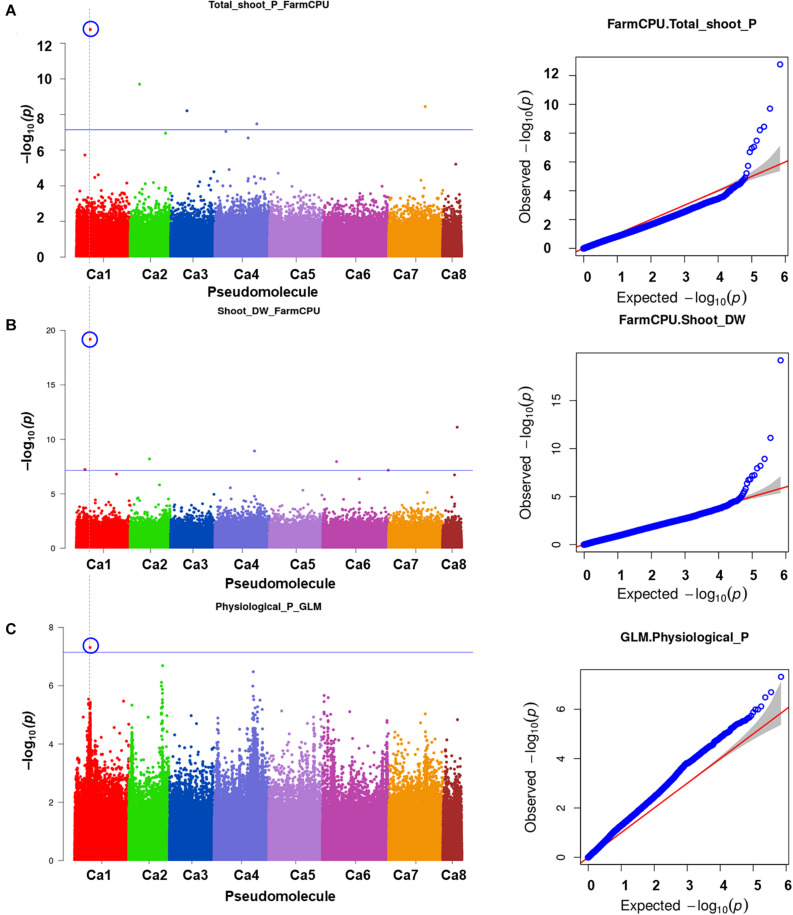
An SNP locus (Ca1_12310101) on Ca1 showing association with three traits **(A)** physiological phosphorus (P)-use efficiency, **(B)** shoot dry weight, and **(C)** shoot P content. Three different statistical models GLM, MLM, and FarmCPU were used to identify the MTAS. The significant MTAs were determined using Bonferroni correction (Table 1).

A total of 10 significant MTAs (three based on GLM and seven based on MLM) for shoot P content—six on Ca4, two on Ca6 and one each on Ca7 and Ca2 were identified (Figure 1A). Two significant MTAs on Ca4 (Ca4_38518152 and Ca4_8269508) were identified with the GLM and MLM models (Supplementary Table 1). The 15 MTAs identified—14 from MLM and one from FarmCPU—explained 5–21% of the phenotypic variation. Five of the MTAs were identified on Ca6 followed by Ca7 (4), Ca4 (2), Ca2 (2), Ca1 (1), and C3 (1) (Figure 1B). A SNP locus, Ca7_33808891, associated with physiological P-Use efficiency present in gene Ca_16189 on Ca7 explained 20.23% PVE (Figure 1C; Supplementary Table 1). A SNP locus, Ca4_38518152, associated with Shoot phosphorus content present in gene Ca_13110 on Ca4 explained 31.5% PVE (Figure 1D; Supplementary Table 1). In the case of physiological PUE one significant MTA (Ca1_12310101) on Ca1 explaining 12.0% of the phenotypic variation was identified. A single nucleotide polymorphism locus (Ca1_12310101) on Ca1 associated with three traits, i.e., physiological P-use efficiency, shoot dry weight, and shoot P content was identified (Figure 2).

### GWAS Signals for Proxy Traits

**FIGURE 3:**
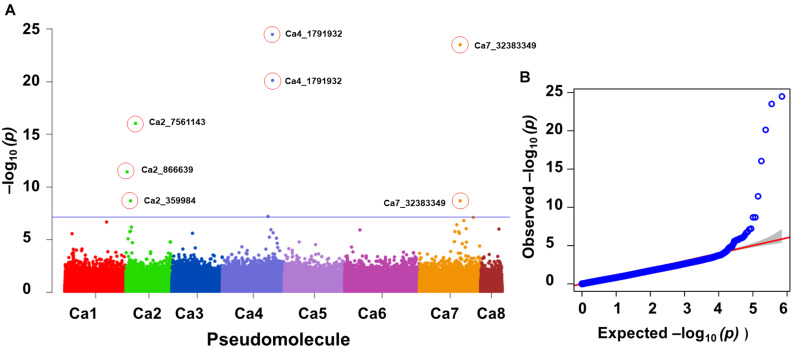
GWAS signal for manganese concentration in mature leaves. Of seven MTAs identified, three on Ca2 were unique and two each on Ca4 and Ca7 were the same SNP loci associated with the trait at different significance levels. **(A)** Manhattan plot showing significant MTAs and **(B)** Q-Q plots for the trait. Three different statistical models GLM, MLM, and FarmCPU were used to identify the MTAS. The significant MTAs were determined using Bonferroni correction (Table 1).

One significant MTA for P concentration in mature leaves (P_ML; mg g^–1^) was identified on Ca4. Seven significant MTAs for Mn concentration in mature leaves were identified on Ca2 (3), Ca4 (2), and Ca7 (Figure 3). The SNP loci on Ca7 (Ca7_32383349) and Ca4 (Ca4_1791932) were associated with manganese concentration in mature leaves with two different *p*-values (Figure 3), while three SNPs associated with the Mn_ML on Ca2 (Ca2_7561143, Ca2_866639, and Ca2_359984). One MTA each for citric (μmol plant^–1^), Ci, carboxylate conc (μmol g^–1^ root dw), malonic (μmol plant^–1^), Pn_area (μmol m^–2^s^–1^), Pn_mass (μmol g^–1^ s^–1^), specific rhizosheath weight (g g^–1^root DW) were identified using FarmCPU. No significant MTAs were identified for these traits using GLM and MLM models.

## Discussion

Understanding the genetics of traits associated with enhanced PUE, including P-acquisition efficiency and P-use efficiency, is essential for its manipulation. In our earlier studies on chickpea, we gained insight into root system architecture, shoot/root traits associated with P-acquisition efficiency and P-use efficiency, and associated proxy traits. Many SNPs associated with root-hair length in P-sufficient and P-deficient conditions have been reported recently (Kohli et al., 2020); here we report SNPs associated with PUE and P-acquisition efficiency related root traits as well as proxy traits, which can be deployed for breeding.

As population structure is important for avoiding spurious associations, we identified three subpopulations using ADMIXTURE in the reference set (233 genotypes) and a smaller subset (91 genotypes). Similarly, earlier studies reported three subpopulations using different marker systems (Thudi et al., 2014) and genome-wide SNP markers (Varshney et al., 2019). In the smaller subset of 91 genotypes, we also identified three subpopulations (Supplementary Figures 1A–D). Four subpopulations were recently reported in a diverse set of 186 genotypes (including 20 Iranian landraces and 166 Kabuli advanced breeding lines from ICRISAT and ICARDA) using DArTseq markers (Farahani et al., 2019). Two subpopulations were reported in a set of 92 (77 landraces and five elite cultivars) chickpea germplasm lines that represent arid, semi-arid, and tropical climates using 8,113 genotyping-by-sequencing based SNPs (Sani et al., 2017).

Three statistical models (GLM, MLM, and FarmCPU) were used to identify genome-wide association signals for root architectural traits, P-acquisition efficiency and PUE-related traits. Similarly, a recent study used different statistical models to compare selected traits with different heritabilities in soybean and maize—the FarmCPU model provided a closer number of QTL than those in the literature and known genomic regions (Kaler et al., 2020). In this study, identification of significant MTAs and determining their usefulness for chickpea improvement was a major focus, rather than comparative utilities of different statistical models. In the present study, significant MTAs for 17 of the 30 root traits analyzed was reported. Earlier, in chickpea, MTAs for drought-tolerance-related root traits were based on screening a reference set in polyvinyl chloride pipes or semi-automated root screening facility at ICRISAT (Thudi et al., 2014; Varshney et al., 2019). In this study, MTAs for root traits studied under semi-hydroponic conditions and the role of root and shoot traits in P-acquisition efficiency and PUE are reported.

The MTA (Ca1_4716136) identified for RGR, is present in a gene (Ca_00555) that encodes a receptor-like cytosolic serine/threonine-protein kinase RBK2 involved in protein phosphorylation in *Arabidopsis thaliana*^1^. Rac-like GTP-binding protein (ARAC5), with 87.8% homology, is localized on the plasma membrane of *Arabidopsis thaliana* root tips. The Ca_01156 gene encodes Acyl-CoA-binding domain-containing protein 4; in *Arabidopsis thaliana*; overexpression of Acyl-CoA -binding protein 3 (ACBP3) results in leaf senescence (Xiao et al., 2010). In chickpea, the pectinesterase gene was downregulated in the roots of salt-tolerant genotypes (Kaashyap et al., 2018). Pectinestarases also play a key role in root-hair initiation and elongation (Cosgrove, 2016). Similarly, another MTA on Ca1 (Ca1_8712480) explained about 20% of the phenotypic variation detected in the GLM and MLM models. The MTA was present in the Ca_02941 gene that encodes 4-coumarate–CoA ligase 1. In tobacco, root length increased by 64% compared with the wildtype, on overexpression of *Fm4CL-like 1* [4-coumarate:CoA ligase 4 (*4CL-like 1*) from *Fraxinus mandshurica*] under mannitol-simulated drought stress (Chen et al., 2019).

Two MTAs (Ca4_38518152 and Ca4_8269508) for shoot P content were present in two genes, Ca_13110 and Ca_08315, which encode NAD kinase 2, chloroplastic-like isoform X2 and piezo-type mechanosensitive ion channel homolog isoform X2, respectively. NAD kinase 2 is involved in phosphorylation. In general, NADK genes show tissue specificity in expression. In *Arabidopsis*, *NADK2* is expressed in leaves, while *TaNADK2* is highly expressed in wheat pistils, caryopses, and endosperm during the reproductive stage (Li et al., 2018). All MTAs for shoot P content were robust and explained 27.7–41.4% of the phenotypic variation. QTL for shoot P content and PUE were located on chromosomes 3 and 4, respectively (Hammond et al., 2009). Of the five MTAs on Ca6, two were in the same gene (Ca_10411), encoding dynamin-related protein 1C. The MTA identified in the FarmCPU model, Ca1_16163105 on Ca1, was present in the Ca_06938 gene that encodes organic cation/carnitine transporter 4-like (OCT), which is involved in homeostasis in animals and has been well-studied in *Arabidopsis thaliana*—disruption of *AtOCT1* affects root development (Lelandais-Brière et al., 2007). Four major pathways in *Lupinus albus* that contribute to PUE are carbon fixation, cluster-root formation, soil P mobilization, and cellular P reuse (Xu et al., 2020). A recent effort to understand the genetic basis of photosynthesis and PUE affecting yield reported that three major QTL (*q14-2, q15-2*, and *q19-2*) explained 6.6–58.9% of the phenotypic variation (Li et al., 2016). Furthermore, the gene that encodes purple acid phosphatase within the *q19-2* region (*Glyma.19G193900*) is a potential candidate for regulating both soybean PUE and photosynthetic capacity. A total of 159 QTNs within 31 genomic regions and genes associated with photosynthesis-related traits under P stress conditions were genotyped in 2,019 soybean accessions using 292,035 high-quality SNPs and phenotyped under adequate- and low-P conditions for 2 years (Lü et al., 2018). The MTA (Ca1_12310101) for physiological PUE is present in a gene that encodes fasciclin-like arabinogalactan protein 12 (Ca_02521); in *Arabidopsis*, this gene is involved in cell wall biogenesis (Figure 2C). Further, this SNP locus was associated with total shoot P content and shoot DW in different models (Supplementary Table 1 and Figure 2). Such shared associations were also reported using different models in mungbean PUE (Reddy et al., 2020). A recent study reported that low expression of selected fasciclin-like arabinogalactan protein genes led to kernel abortion in maize (*Zea mays*) and *Arabidopsis thaliana* seeds (Cagnola et al., 2018). In cereals such as rice, SNP loci on chromosomes 1, 4, 11, and 12 are associated with PUE (Wissuwa et al., 2015).

Pang et al. (2018a) reported that root foraging and root physiology, such as the exudation of carboxylates into the rhizosphere, are important strategies for plant P acquisition efficiency. A positive correlation was also identified between mature leaf Mn concentration and rhizosheath carboxylate amount relative to root DW, and hence the carboxylate-releasing P-mobilizing strategy was proxied by foliar Mn concentration in a large set of chickpea germplasm under low P supply (Pang et al., 2018a). The MTA for Mn concentration in mature leaves identified on Ca4 was in gene Ca_14893 that encodes zinc finger BED domain-containing protein RICESLEEPER 2-like protein. Carboxylate exudation is an important physiological root trait that enables plants to mine soil P (Lambers et al., 2008, 2015; Richardson and Simpson, 2011). Carboxylate concentrations in the rhizosheath are positively correlated with shoot P content (Pang et al., 2018a). A SNP locus associated with carboxylate amount was identified in the rhizosheath (μmol g^–1^ root DW) associated with a Ca_11019 gene that encodes for ABC1 family protein, and is an integral part of the membrane. The FarmCPU model identified two significant MTAs (Ca4_15651907 and Ca8_230753) in RhizoPH.

## Conclusion

In summary, the SNP loci associated with more than one trait were identified. For instance, Ca1_12310101 on Ca1 is associated with three traits (i.e., physiological PUE, shoot DW, and shoot P content), Ca2_31290805 on Ca2 is associated with P utilization and total root length, Ca4_37796452 on Ca4 is associated with RMR and shoot P content, Ca3_6798755 on Ca3 is associated with RGR and TRLz1, and Ca7_5414752 on Ca7 is associated with leaf intracellular CO_2_ concentration and WUE. The MTA for Mn in mature leaves identified on Ca4 was in gene Ca_14893 that encodes zinc finger BED domain-containing protein RICESLEEPER 2-like protein. The MTAs reported in this study can be used in chickpea breeding programs to enhance PUE.

## Data Availability Statement

Publicly available datasets were analyzed in this study. This data can be found here: CNSA (https://db.cngb.org/cnsa/) of CNGBdb with accession code CNP0000370.

## Author Contributions

KHMS and RKV conceived the study. MT and DK performed GWAS. YC and JP generated the phenotyping data. PB, MRo, AC, MRy, and HL contributed to the resources and the writing. MT, RKV, and KHMS prepared the manuscripts. All authors read and approved the final manuscript.

## Conflict of Interest

The authors declare that the research was conducted in the absence of any commercial or financial relationships that could be construed as a potential conflict of interest.

## Footnotes

1 https://www.uniprot.org/uniprot/Q8RXC8

## Supplementary Material

Supplementary Figure 1 Example of spurious associations detected for taproot length (TRL, mm) in the GLM model, with no associations detected in the MLM and FarmCPU models.

Supplementary Figure 2 Population structure. **(A,B)** Three sub-populations among 233 genotypes phenotyped for root and phosphorus-related traits, and **(C,D)** three sub-populations among 91 genotypes phenotyped for shoot/root morphological and physiological traits. Each colored vertical line represents proportions of ancestral populations (K) for each individual. Optimum K value determined using ADMIXTURE’s cross-validation procedure.

Supplementary Table 1 Details of significant MTAs with different traits and functional annotation of SNP loci.
