# Supplementary figures and images for: Novel Genes and Genetic Loci Associated With Root Morphological Traits, Phosphorus-Acquisition Efficiency and Phosphorus-Use Efficiency in Chickpea
Source: Front Plant Sci. 2021 May 28;12:636973. doi: 10.3389/fpls.2021.636973 (PMC8192852; doi:10.3389/fpls.2021.636973)

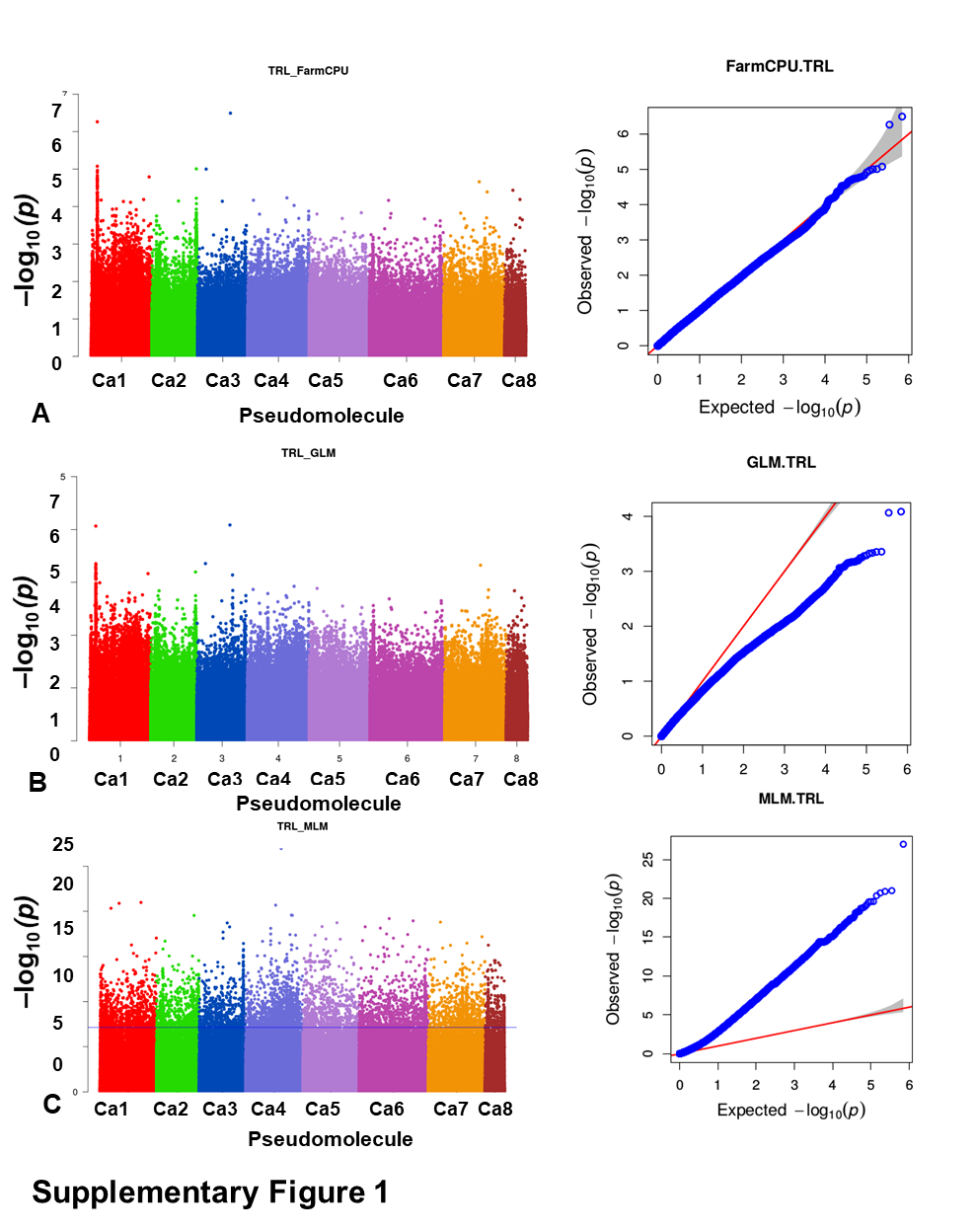

Supplement: Supplementary Figure 1 — Example of spurious associations detected for taproot length (TRL, mm) in the GLM model, with no associations detected in the MLM and FarmCPU models. [file Image_1.TIF]

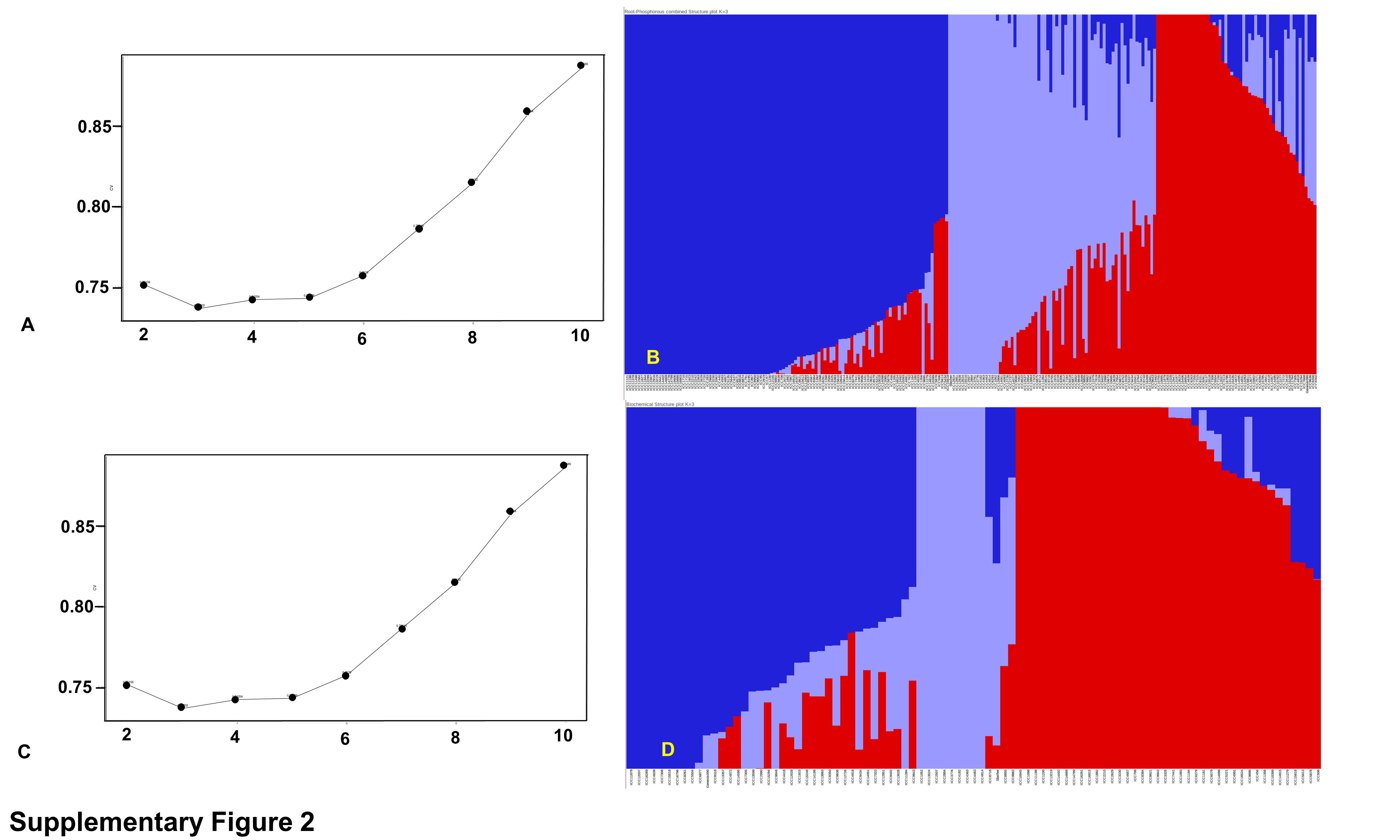

Supplement: Supplementary Figure 2 — Population structure. (A,B) Three sub-populations among 233 genotypes phenotyped for root and phosphorus-related traits, and (C,D) three sub-populations among 91 genotypes phenotyped for shoot/root morphological and physiological traits. Each colored vertical line represents proportions of ancestral populations (K) for each individual. Optimum K value determined using ADMIXTURE’s cross-validation procedure. [file Image_2.TIF]
